# Metabolic engineering strategies for optimized lignan production in plants

**DOI:** 10.3389/fpls.2026.1827862

**Published:** 2026-06-03

**Authors:** Sangchul Choi, Saet Buyl Lee, Beom-Gi Kim

**Affiliations:** Plant Biomaterials and Biotechnology Division, National Institute of Agricultural Sciences, Rural Development Administration, Jeonju, Republic of Korea

**Keywords:** lignan, metabolic engineering, plant secondary metabolites, podophyllotoxin, sesamin, synthetic biology

## Abstract

Plant secondary metabolites, including lignans, play essential roles in plant defense and adaptation, and their pharmacological properties are increasingly valued for human health. Lignans are dimers derived from the phenylpropanoid pathway whose biosynthesis is tightly controlled by dirigent proteins, laccases, and other redox-related enzymes. Recent advances in plant metabolic engineering have progressed from simple single-gene overexpression to integrated strategies that combine transcriptional regulation, metabolic flux optimization, and CRISPR-based genome editing. This review proposes the “Push-Pull-Release” framework to organize these approaches. This framework utilizes three complementary mechanisms: “Push” strategies to increase precursor supply through master transcription factors such as AtMYB85 and enzymatic overexpression; “Pull” methods to redirect flux by attenuating competing metabolic sinks, including CHS- and F5H-associated branches; and “Release” mechanisms that alleviate intrinsic pathway repression by targeting MYB repressors and post-translational regulators such as KFB proteins. Advanced control is further achieved through synthetic biology principles, including modular pathway reconstruction, multiplex genome editing, and spatiotemporal regulation through tissue-specific, inducible, and subcellular engineering strategies. Case studies on sesamin, podophyllotoxin, and a proposed SDG production framework in flax illustrate how these multi-layered strategies may be integrated in plant systems. However, maximizing lignan yield must be balanced against trade-offs in plant structural integrity and disease resistance. Accordingly, future lignan metabolic engineering should integrate multi-layered controls with spatiotemporal regulation and systematic phenotypic evaluation to achieve sustainable production while preserving plant fitness.

## Introduction

Secondary metabolites play crucial roles in plant defense against pathogens and herbivores, environmental stress adaptation, and ecosystem signaling (Dong and Lin, 2021). These plant-derived compounds are highly valued in human health and industry, providing essential therapeutic and functional biomaterials (Jang et al., 2022; Koyama et al., 2022; Van Beirs et al., 2025). Lignans are a distinct class of phenylpropanoid-derived secondary metabolites, generally defined as dimers of two phenylpropanoid (C_6_–C_3_) units formed through oxidative coupling of monolignol precursors. Among the possible interunit linkages, the *β*–*β′* (8–8′) C–C bond is the most prevalent and is exemplified by pinoresinol, secoisolariciresinol, and lariciresinol (Koyama et al., 2022b; Satake et al., 2013, 2015). In addition to this canonical linkage, lignans can also arise through alternative coupling patterns, including *β*–5′ (8–5′), *β*–*O*–4′ (8–*O*–4′), and 5–5′ interunit linkages, thereby generating a structurally diverse array of compounds with distinct ring systems, stereochemical configurations, and biological activities (Saleem et al., 2005; Teponno et al., 2016). Unlike high-molecular-weight lignin polymers, lignans consist of low-molecular-weight compounds with diverse isomeric forms and biological functions (Koyama et al., 2022b; Han et al., 2024; Feduraev et al., 2021).

**Figure 1:**
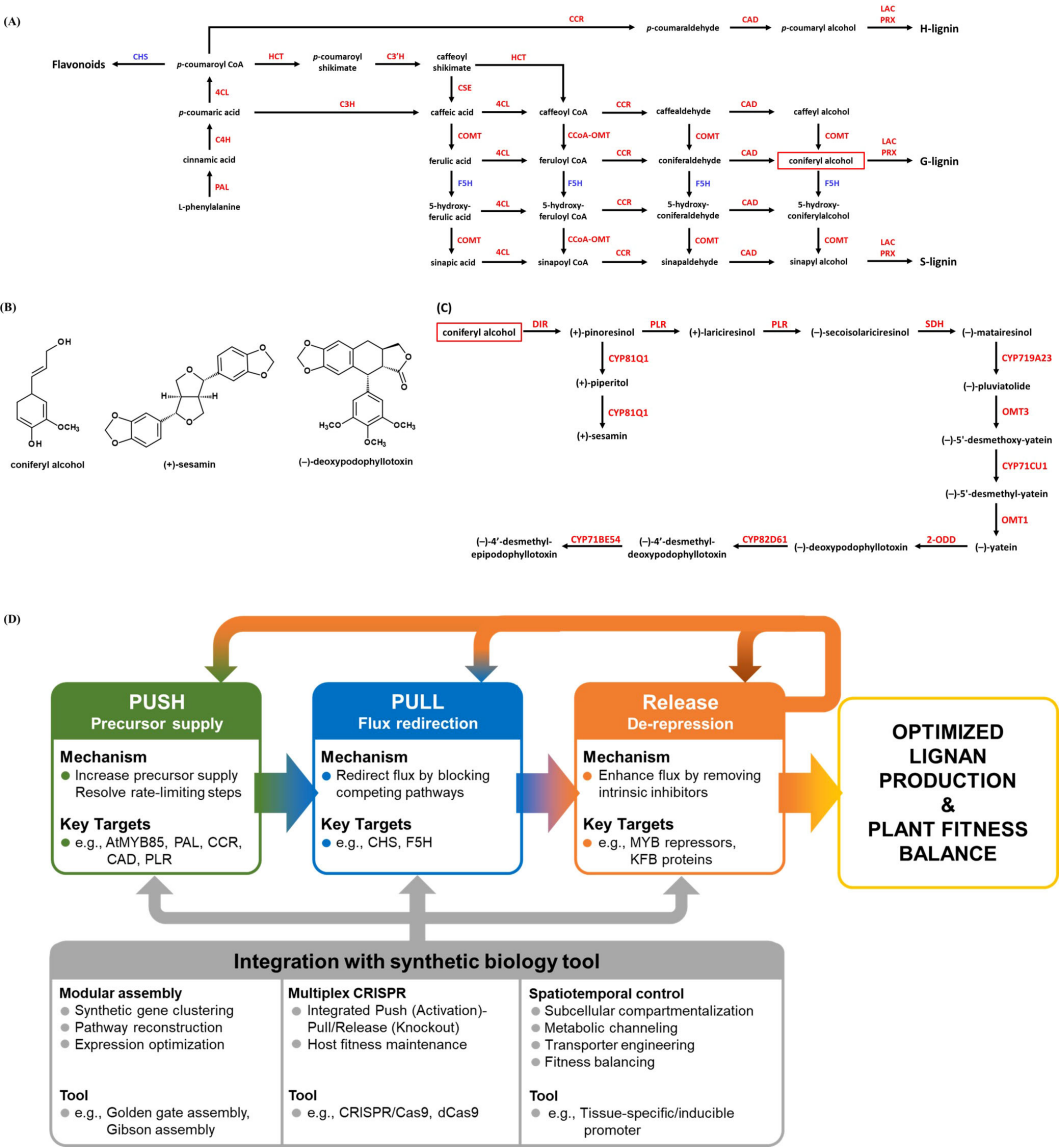
Integrated overview of lignan biosynthesis and “Push-Pull-Release” strategies for plant lignan metabolic engineering. **(A)** Overview of the phenylpropanoid pathway leading to flavonoids, lignin, and lignan precursors. Sequential enzymatic conversions start from L-phenylalanine and branch into flavonoid biosynthesis and monolignol production for lignin monomer (H-, G-, and S-lignin). The principal enzymes and end products depicted are as follows: PAL, phenylalanine ammonia-lyase; C4H, cinnamate 4-hydroxylase; 4CL, 4-coumarate:CoA ligase; CHS, chalcone synthase; HCT, hydroxycinnamoyl-CoA:shikimate hydroxycinnamoyl transferase; C3’H, *p*-coumaroyl shikimate 3’-hydroxylase; CSE, caffeoyl shikimate esterase; C3H, coumarate 3-hydroxylase; COMT, caffeic acid O-methyltransferase; F5H, ferulate 5-hydroxylase; CCoA-OMT, caffeoyl-CoA O-methyltransferase; CCR, cinnamoyl-CoA reductase; CAD, cinnamyl alcohol dehydrogenase; LAC, laccase; PRX, peroxidase; H-lignin, hydroxyphenyl-lignin; G-lignin, guaiacyl lignin; S-lignin, syringyl-lignin. The pathway also highlights interconnections leading to lignan biosynthesis. Enzyme names are shown at each reaction step, and the main product classes are indicated at pathway termini. **(B)** Chemical structures of monolignol precursor (coniferyl alcohol) and representative lignans derived from phenylpropanoid pathway. **(C)** Biosynthetic pathway from coniferyl alcohol to sesamin and podophyllotoxin derivatives. This panel illustrates the enzymatic conversion steps starting from coniferyl alcohol as the precursor of lignan biosynthesis. Key enzymes and their full names are as follows: DIR, dirigent protein; PLR, pinoresinol-lariciresinol reductase; SDH, secoisolariciresinol dehydrogenase; CYP81Q1, (+)-piperitol/(+)-sesamin synthase; CYP719A23, (–)-pluviatolide synthase; OMT3, (–)-pluviatolide-O-methyltransferase; CYP71CU1, (–)-5’-desmethoxy-yatein hydroxylase; OMT1, (–)-5’-desmethyl-yatein O-methyltransferase; 2-ODD, 2-oxoglutarate/Fe(II)-dependent dioxygenase; CYP82D61, (–)-deoxypodophyllotoxin 4-hydroxylase; CYP71BE54, (–)-4’-desmethyl-deoxypodophyllotoxin hydroxylase. Each step highlights the enzyme responsible for the specific biochemical reaction (Gasper et al., 2016; Lau and Sattely, 2015; Yao et al., 2021). **(D)** Schematic representation of the “Push-Pull-Release” framework for lignan metabolic engineering. “Push” promotes precursor supply, “Pull” redirects flux by blocking competing pathways, and “Release” removes negative regulators to relieve pathway repression. These strategies are linked with synthetic biology tools for modular assembly, multiplex CRISPR, and spatiotemporal control to support lignan production and plant fitness (See **Table 1** for a comparative overview of major engineering strategies and their risks and considerations).

Lignan biosynthesis initiates from the monolignol precursor coniferyl alcohol (CA), undergoing oxidative dimerization mediated by dirigent proteins, laccases, and redox enzymes to produce structurally diverse compounds (Satake et al., 2015; Chen et al., 2024; Li et al., 2022). This pathway is stringently regulated in a spatiotemporal manner, influenced by species, tissue specificity, and environmental factors (**Figure 1**) (Dong and Lin, 2021; Satake et al., 2013; Rohde et al., 2004; Liu et al., 2021). Lignans exhibit diverse pharmacological activities including potent antioxidant, anticancer, anti-inflammatory, antiviral, cardioprotective, and neuroprotective effects, attracting growing attention for pharmaceutical applications and chronic disease prevention (Jang et al., 2022; Koyama et al., 2022).

Beyond their pharmacological relevance, lignans play important roles in plant defense, as dirigent protein-mediated stereoselective monolignol coupling is induced in response to pathogen attack and wounding, leading to the accumulation of antimicrobial lignans such as pinoresinol and related compounds that contribute to resistance against microbial invasion, while yatein-related lignans have also been implicated in primed defense responses to necrotrophic fungi (Li et al., 2017; Sanmartín et al., 2020). From an industrial perspective, several lignans also possess substantial commercial value. Secoisolariciresinol diglucoside (SDG) from flaxseed (*Linum usitatissimum*) is widely recognized as a functional food and nutraceutical ingredient owing to its phytoestrogenic and antioxidant properties, whereas (−)-podophyllotoxin, isolated from Himalayan mayapple (*Sinopodophyllum hexandrum*), serves as the indispensable chemical scaffold for the semi-synthetic anticancer agents etoposide and teniposide that are used clinically worldwide (Satake et al., 2013; Lau and Sattely, 2015). Collectively, these defense-related, health-promoting, and industrially relevant functions underscore the compelling rationale for developing scalable lignan production platforms through metabolic engineering.

Despite their valuable properties, natural lignan production faces significant challenges due to low yield, complex regulation, and limited accessibility from native sources. Recent advances in plant metabolic engineering, synthetic biology, and genome editing offer promising routes to enhance lignan biosynthesis through multi-layered engineering strategies. The following sections survey these integrated approaches under “Multi-layered engineering strategies for lignan production enhancement”, systematically examining Precursor supply optimization (“Push”), Metabolic flux redirection (“Pull”), Regulatory control liberation (“Release”), and Advanced synthetic biology approaches to achieve sustainable high-level production while maintaining plant viability (as shown in **Figure 1D**).

The “Push-Pull-Release” framework proposed in this review organizes these strategies as an integrated system. Unlike the “Push-Pull-Block” framework in conventional metabolic engineering, which focuses on blocking competitive pathways through gene knockouts, the “Push-Pull-Release” strategy refines this concept for plant systems. It incorporates a “Release” component to target intrinsic transcriptional repressors and post-translational regulators, thereby de-repressing the target pathway—a regulatory layer particularly prominent in the sophisticated specialized metabolism of plants.

## Precursor supply optimization: the “Push” strategy

### Enzymatic step reinforcement

The most straightforward strategy involves the constitutive or inducible overexpression of key biosynthetic enzymes, pushing metabolic flux toward desired final products. This strategy addresses pathway bottlenecks by increasing enzyme activity at rate-limiting steps.

Key biosynthetic enzymes spanning phenylpropanoid pathway entry to terminal lignan-specific steps have been systematically targeted. Phenylalanine ammonia-lyase (PAL), located at the phenylpropanoid pathway entry point, is a primary rate-limiting enzyme determining carbon flux into the pathway. Studies in wheat (*Triticum aestivum*) demonstrated that *PAL* overexpression significantly boosted phenylpropanoid pathway activity and lignin accumulation (Han et al., 2024; Feduraev et al., 2021), while *Arabidopsis thaliana* mutant analysis revealed *PAL* isoforms regulate lignin and phenylpropanoid metabolite pools (Rohde et al., 2004; Huang et al., 2010).

Downstream, cinnamoyl-CoA reductase (CCR) catalyzes a crucial monolignol formation step. *CCR* overexpression in *Brassica napus* substantially increased lignin accumulation and monolignol precursor availability, establishing CCR as a key regulatory point (Liu et al., 2021). Similarly, cinnamyl alcohol dehydrogenase (CAD), catalyzing the final monolignol biosynthesis step, directly influences CA availability; *CAD* overexpression in *Populus* increased total lignin and lignan precursor levels (Bagniewska-Zadworna et al., 2014).

At lignan structural diversification stages, *pinoresinol-lariciresinol reductase* (*PLR*) overexpression substantially boosts lignan accumulation and diversity. Flax (*L. usitatissimum*) *PLR* upregulation markedly increased total lignan content (Hano et al., 2006), while heterologous *PLR* and *secoisolariciresinol dehydrogenase* (*SDH*) overexpression in tobacco (*Nicotiana benthamiana*) drove dramatic increases in matairesinol and related lignans (Schultz et al., 2019; Lau and Sattely, 2015).

Product stability critically affects productivity. UDP-glucosyltransferase (UGT), attaching sugar residues to form stabilizing glycosides, substantially enhances intracellular storage. In sesame (*Sesamum indicum*), *UGT* gene expression promotes stepwise sesaminol glucosylation, increasing stable lignan storage forms (Ono et al., 2020).

However, single enzyme overexpression has limitations: excessive expression can create new bottlenecks or cause harmful intermediate accumulation. This points to necessity of more integrated strategies mimicking sophisticated plant regulatory networks (**Table 1**).

**Table 1:** Comparative analysis of key metabolic engineering strategies for enhancing lignan production.

Strategy	Mechanism of action	Key targets	Relative complexity	Potential for yield increase	Major risks and considerations
Single Enzyme Overexpression	Increases flux through specific rate-limiting step (“Push”)	PAL, CCR, CAD, PLR	Low	Medium	Potential for new bottlenecks; risk of intermediate toxicity
Transcription Factor Overexpression	Coordinated upregulation of entire pathway (“Master Push”)	MYB, ERF activators	Medium	High to Very High	Possible significant metabolic burden; well-characterized TFs needed
Competing Pathway Suppression	Blocks metabolic outlets to redistribute precursor flow (“Pull”)	CHS, F5H	Medium (RNAi) to High (CRISPR)	Medium to High	Possible negative effects on plant growth and development (e.g., lignin reduction)
Negative Regulator Removal	Removes inherent transcriptional or post-translational brakes (“Release”)	MYB repressors, KFB proteins	High	High	Risk of uncontrolled metabolic flux causing toxicity; difficult to predict effects
Pathway Reconstruction	Assembly of multi-gene pathways in heterologous hosts	Entire pathways (e.g., etoposide)	Very High	Very High	Technically very challenging; requires optimization of enzyme ratios and host compatibility
Subcellular Organelle Engineering	Spatial and temporal optimization of enzymes and transporters	Signal peptides, transporters, promoters	High	Enhances effectiveness of other strategies	Requires detailed knowledge of protein localization and transport mechanisms.

### Master transcriptional regulation

Beyond individual enzyme control, simultaneously and harmoniously activating multiple pathway genes via transcription factors (TFs) represents advanced metabolic engineering. Plants utilize MYB, WRKY, NAC, and Ethylene response factor (ERF) TFs as “master switches” coordinating entire metabolic pathway activation. This approach alleviates multiple bottlenecks simultaneously, much like a conductor synchronizing orchestra sections.

MYB family TFs are extensively studied lignan and lignin biosynthesis regulators. *Arabidopsis MYB85* overexpression induces monolignol and lignan accumulation (Geng et al., 2020; Kim et al., 2022), while *Betula platyphylla MYB46* overexpression broadly activates lignin biosynthetic genes (Guo et al., 2017; Ko et al., 2014). Similarly, *Arabidopsis MYB58* and *MYB63* overexpression achieves full pathway activation with massive compound accumulation (Zhou et al., 2009).

WRKY and NAC family TFs also regulate secondary metabolism. WRKY TFs regulate phenylpropanoid genes responding to environmental stresses, promoting lignan biosynthesis (Markulin et al., 2019; Xiao et al., 2020), while NAC TFs, master secondary cell wall regulators, cause widespread lignin and lignan biosynthesis activation (Golfier et al., 2017).

Strong evidence emerges from ERF family TF Lignan biosynthesis-associated factor 1 (LTF1) studies. *LTF1* overexpression in *Isatis indigotica* hairy root increased *dirigent protein 1* (*DIR1*) expression 34-fold, yielding 2- to 5-fold increases in various lignans, with pinoresinol rising approximately 5-fold. Stress response modulation of LTF1 coordinately enhances biosynthesis and plant defense (Chen et al., 2024), positioning ERF TFs as promising targets for developing stress-tolerant, high-yield crops.

Crucially, MYB family functional differentiation exists: some members act as activators while others serve as repressors. AtMYB85 functions as a strong activator, while R2R3-MYB subgroup 4 members (MYB4, MYB7, and MYB32) act as negative regulators, binding promoters of key genes like *PAL*, *cinnamate 4-hydroxylase* (*C4H*), *4-coumarate:CoA-ligase* (*4CL*), and *CAD*, repressing their transcription (Jin et al., 2000). This dual nature enables combined strategies of activator overexpression with repressor suppression.

Master regulator potential is clearly demonstrated by podophyllotoxin, a complex anticancer compound. Its multi-step pathway makes simple heterologous gene introduction insufficient for high production (Lau and Sattely, 2015; Schultz et al., 2019). Early attempts introducing ten podophyllotoxin biosynthetic genes into tobacco achieved very low yields due to limited CA supply (Lau and Sattely, 2015; Schultz et al., 2019). The breakthrough arose when *AtMYB85* was co-expressed with pathway genes in tobacco, AtMYB85 reactivated endogenous tobacco monolignol biosynthesis comprehensively, “reprogramming” host plant metabolism (the pathway shown in **Figure 1C**). This yielded remarkable production levels: approximately 1 mg/g dry weight (DW) etoposide aglycone and 35 mg/g DW (−)-deoxypodophyllotoxin (DPT), representing roughly 100-fold and 8-fold increases compared to precursor feeding or native Himalayan mayapple plants. Undesired glycosylated byproducts were simultaneously suppressed, improving purity (Kim et al., 2022). This case clearly illustrates that intelligently leveraging plant-evolved regulatory networks is far more efficient than forcibly manipulating single enzymes, suggesting future metabolic engineering will transcend gene insertion toward rewiring intrinsic plant regulatory circuits for optimized production.

## Metabolic flux redirection: the “Pull” strategy

Lignan biosynthesis relies on phenylpropanoid pathway precursors, which also feed competing secondary metabolites like lignin and flavonoids. These branched pathways act as metabolic “sinks”, diverting resources from lignan production (**Figure 1A**). Suppressing competing route enzymes via RNA interference (RNAi) and Clustered Regularly Interspaced Short Palindromic Repeats (CRISPR) genome editing effectively redirects flux toward target pathways.

RNAi-mediated *PLR* knockdown in *Forsythia koreana* cell cultures, blocking pinoresinol conversion, caused over 20-fold pinoresinol glycoside accumulation compared to controls (Kim et al., 2009). In poplar (*Populus tremula × P. alba*), *4CL* knockout, a pivotal lignin branch point enzyme, altered lignin composition and caused phenylpropanoid metabolite accumulation (Tsai et al., 2020). Similarly, *ferulate 5-hydroxylase* (*F5H*) knockout in *B. napus*, essential for syringyl (S)-lignin biosynthesis, resulted in decreased S-lignin content and enhanced phenylpropanoid precursor flux (Cao et al., 2022).

Particularly notable is *chalcone synthase* (*CHS*) knockout, the flavonoid pathway entry enzyme consuming *p*-coumaroyl-CoA, a major metabolic sink. Deleting *CHS* diverts more precursor toward monolignol and lignan biosynthesis (Li et al., 2010; Wang et al., 2025). In *Vitis davidii*, *CHS2* knockout reduced flavonoid accumulation but increased stilbenoids like resveratrol 4.1-fold, confirming successful substrate redistribution (Lai et al., 2025). Lignin pathway blocking is also highly effective: *F5H* knockout inhibits S-lignin formation, resulting in guaiacyl (G)-lignin precursor CA accumulation (Cao et al., 2022; Wu et al., 2019), potentially enhancing lignan biosynthesis precursor availability.

Interestingly, flux redirection toward lignans can be achieved by reinforcing target pathway enzymes rather than only blocking competitors. Overexpressing *dirigent protein-related 11* (*PtDIR11*) in *P. trichocarpa* increased flavonoid and lignan levels 2.28- and 1.67-fold, respectively, without altering total lignin, suggesting PtDIR11 preferentially directs shared precursors toward lignans (Li et al., 2022). Metabolic resource competition is thus a dynamic equilibrium manipulated by both enhancing target routes and weakening competing pathways.

However, strategic interventions must consider critical trade-offs. Excessive lignin biosynthesis suppression—a vital structural polymer—may compromise plant viability. Hence, tissue-specific or inducible promoters restricting pathway modulation spatially or temporally are essential for balancing productivity with host plant health (See “Major Risks and Considerations” column in **Table 1**) (Xie et al., 2018).

## Regulatory control liberation: the “Release” strategy

Plant metabolic networks employ sophisticated negative regulatory mechanisms acting as metabolic “brakes”, suppressing pathway activity. Advanced metabolic engineering identifies and removes these intrinsic brakes, enabling sustained high pathway activity—analogous to simultaneously pressing the accelerator and releasing the brake for maximum speed.

Transcriptional repression is exemplified by MYB4, MYB7, and MYB32, known repressors directly binding promoter AC elements of *PAL*, *4CL*, *CCR*, and *CAD*, repressing their expression. Evidence from switchgrass (*Panicum virgatum*) and *B. platyphylla* demonstrated that *MYB4* homolog overexpression decreased lignin biosynthetic gene expression, confirming repressive function (Rao et al., 2019; Yu et al., 2020). Consequently, knockout of these repressor genes releases transcriptional brakes: *Arabidopsis atmyb4* mutants accumulate significantly higher sinapoyl malate levels, indicating pathway activation that expands CA pools, promoting lignan biosynthesis (Jin et al., 2000).

Post-translationally, Kelch repeat F-box (KFB) proteins play critical negative regulatory roles. In *Arabidopsis*, KFB1, KFB20, KFB39, and KFB50 form part of the Skp1-Cullin-F-box protein (SCF)-type E3 ubiquitin ligase complexes targeting PAL for ubiquitination and 26S proteasomal degradation. Thus KFB proteins directly regulate PAL stability, acting as metabolic “governors” controlling carbon influx (Zhang et al., 2013). *KFB* gene knockout reduces PAL degradation, increasing PAL stability and activity, substantially boosting phenylpropanoid pathway carbon flow. Higher lignin content in *KFB* mutants indicates elevated CA biosynthesis and availability (Zhang et al., 2015). Since lignans derive from CA dimers, *KFB* knockout likely effectively enlarges precursor pools for enhanced lignan production.

Plant metabolic networks are finely tuned by multi-layered control: activators (accelerator pedals), repressors (brakes), and protein degradation machinery (governors). Integrated metabolic engineering must consider all three control points. Combining activator overexpression like *AtMYB85* with repressor knockout such as *MYB4* in “Push-and-Release” approaches induces pathway activation beyond physiological limits. While this multi-layered strategy opens new horizons, it carries risks of metabolic overload and cytotoxicity, demanding precise balanced regulation.

## Advanced synthetic biology approaches for lignan production

### Pathway reconstruction through modular engineering

Synthetic biology aims to redesign metabolic pathways using engineering design principles embracing modularity, spatial organization, and multi-layered control. Nature often clusters metabolic pathway genes on chromosomes, ensuring simultaneous, balanced expression maintaining smooth flux while preventing toxic intermediate accumulation. Synthetic biology artificially assembles functionally related genes into synthetic clusters on single expression vectors (Bharadwaj et al., 2021).

Reconstruction of the etoposide aglycone biosynthetic pathway exemplifies this strategy’s efficacy. Six essential biosynthetic genes were combinatorially expressed in tobacco using multiple vectors simultaneously, successfully reconstituting a complex multi-step pathway with high target compound production. This exemplifies how artificial gene clustering and combinational expression enable complicated pathway reconstruction (Lau and Sattely, 2015).

Modular cloning techniques like Golden Gate and Gibson assembly facilitate such complex multi-gene constructs, using standardized DNA parts (promoters, genes, and terminators) with defined overlapping ends enabling rapid, precise assembly like building blocks (Bird et al., 2022; Gibson et al., 2009). This modularity allows easy component swapping to optimize gene order and expression levels, crucial for maximizing target metabolite production.

### System-level control through multiplex genome editing

CRISPR/CRISPR-associated protein 9 (Cas9)-based multiplex genome editing revolutionizes metabolic engineering, enabling simultaneous precise control of multiple regulatory genes within metabolic networks, allowing comprehensive system-wide metabolic flux rewiring—previously impossible with single-gene targeting (McCarty et al., 2020).

Multiplex editing’s true power lies in implementing integrated strategies. A single CRISPR system can knock out multiple competing pathway genes creating a “Pull” effect favoring desired flux, while simultaneously employing engineered nuclease-dead Cas9 (dCas9)-based transcriptional activators upregulating target pathway genes effectuating a “Push” (Das et al., 2024; McCarty et al., 2020). This coordinated Push-Pull genome-scale regulation enables high-level target accumulation without severely compromising plant growth or development, overcoming limitations of less precise approaches.

Despite its transformative potential, the deployment of multiple CRISPR modalities within a single plant system presents substantial technical and biological constraints. In particular, the concurrent delivery of multiple gRNAs and Cas9- or dCas9-based effector constructs requires highly efficient multiplexed transformation or vector systems, yet cargo capacity and transformation efficiency remain major bottlenecks that limit the extent of simultaneous editing or regulation achievable in a single event (Lowder et al., 2017; McCarty et al., 2020). Likewise, combining “Pull” strategies such as *CHS* or *F5H* disruption with “Push” strategies mediated by transcriptional activation of *PAL* or *PLR* can redirect metabolic flux, but the resulting redistribution is often spatially and developmentally heterogeneous and may cause transient accumulation of reactive phenylpropanoid intermediates that perturb cellular homeostasis (Vanholme et al., 2012).

Moreover, Cas9-mediated disruption of lignin branch-point genes can compromise structural and developmental programs; for example, perturbation of *F5H* alters S-lignin deposition and modifies lignin chemistry, underscoring the need to balance flux redirection with plant fitness (Ménard et al., 2022). Accordingly, coordinated “Push-Pull-Release” engineering should preferably be implemented using tissue-specific or inducible promoters to confine pathway modulation to tissues where lignan accumulation is desired and structural lignin requirements are less stringent (Xie et al., 2018). Finally, unbiased whole-genome off-target assessment together with compositional and biomechanical phenotyping should be regarded as essential quality-control steps before yield gains are interpreted (Das et al., 2024).

Modular pathway reconstruction and multiplex genome editing convergence offers unparalleled speed, precision, and control, representing metabolic engineering’s cutting edge. This synergy is crucial for developing commercially viable, scalable lignan production platforms.

### Spatial and temporal regulation through subcellular compartmentalization

Cells are highly organized, compartmentalized factories leveraging organelles [endoplasmic reticulum (ER), Golgi apparatus, vacuoles, and plastids], membranes, and transport systems. Advanced metabolic engineering approaches regard cells as structured buildings, leveraging cellular infrastructure to build more efficient production lines, precisely controlling spatial and temporal enzyme, transporter, and metabolite localization (Bar-Peled and Kory, 2022; Huttanus and Feng, 2017).

A fundamental strategy involves attaching signal peptides to biosynthetic enzymes targeting them to specific organelles like plastids or vacuoles. Compartmentalization offers two main advantages: reduced cytotoxicity from accumulated reactive intermediates, and enhanced reaction efficiency through high substrate concentration in designated locations (Huttanus and Feng, 2017; Rozov and Deineko, 2022). As an example of subcellular compartmentalization, targeting carotenoid biosynthetic enzymes to plastids via plastid-targeting signal peptides is important for enabling functional carotenoid biosynthesis and can improve product accumulation relative to mislocalized cytosolic expression, depending on the enzyme and host context (Quian-Ulloa and Stange, 2021).

The ER membrane serves as a strategic site for metabolic channeling through multi-enzyme complexes called metabolons. These physically associate multiple enzymes, facilitating direct intermediate transfer between enzymes, preventing diffusion and loss, enhancing metabolic efficiency (**Figure 1A**) (Biala and Jasinski, 2018; Gou et al., 2018). Several phenylpropanoid pathway enzymes form such complexes, identifying the ER as a central hub of metabolic channeling.

Notably, even subtle adjustments of protein localization affect metabolic profiles. *Arabidopsis* membrane steroid binding proteins (MSBP1 and MSBP2) form scaffold complexes with essential lignin biosynthesis P450 enzymes on the ER membrane, maintaining enzyme stability and activity. Suppression of *MSBP* expression reduces these P450 levels and activities, affecting lignin biosynthesis and the S/G lignin ratio, demonstrating that spatial protein organization is an important biosynthetic pathway regulatory mechanism (Gou et al., 2018).

Beyond enzymes, transporter engineering is critical for subcellular metabolic optimization. ATP-binding cassette (ABC) and Major Facilitator Superfamily (MFS) transporters move lignans and their precursors across membranes (Miao and Liu, 2010; Niño-González et al., 2019). *Arabidopsis* tonoplast-localized transporters actively import monolignol glucosides into vacuoles, effectively regulating cytosolic free monolignol levels (Liu et al., 2011; Miao and Liu, 2010). Norway spruce (*Picea abies*) studies demonstrated that MFS transporters mediate coniferin transport across the tonoplast via a proton antiport mechanism (Väisänen et al., 2020).

These transporters underpin “Push-Pull” metabolic engineering strategies. “Push” entails biosynthetic enzyme overexpression increasing precursor supply, while “Pull” involves specific ABC or MFS transporter overexpression efficiently exporting final products into storage compartments like vacuoles. Simultaneous Push-Pull implementation alleviates feedback inhibition and overcomes pathway bottlenecks, yielding substantially higher production yields than either strategy alone.

Spatial and temporal control extends to regulatory factors themselves. For TFs, nuclear localization is essential; removing nuclear localization signals prevents nuclear entry and target gene activation (Marathe et al., 2024; Zhou et al., 2015). Temporal control is achieved using tissue-specific or inducible promoters to govern the when and where of gene expression. Root-specific promoters enable pathway activation exclusively in roots, reducing whole-plant metabolic burden, while heat-shock inducible promoters allow on-demand gene expression triggering (Forestier et al., 2023; Li et al., 2013).

Such spatial and temporal regulation resolves fundamental production yield versus plant health trade-offs. Restricting potentially burdensome pathway activity to specific tissues or developmental stages achieves high-yield sustainable biological production without compromising overall plant viability.

## Case study: sesamin biosynthesis engineering

Recent advances integrate pathway activation and flux redirection strategies to enhance valuable lignan production. Sesamin biosynthesis engineering began with identifying *S. indicum* cytochrome P450 CYP81Q1, which catalyzes two methylenedioxy bridges converting (+)-pinoresinol to (+)-sesamin (Ono et al., 2006). *Forsythia* cell cultures were engineered suppressing endogenous *PLR* via RNAi, blocking competing (+)-pinoresinol conversion, while introducing sesame *CYP81Q1* establishing novel sesamin biosynthetic routes. This dual approach eliminated native matairesinol formation and achieved remarkable 20-fold pinoresinol precursor accumulation, successfully enabling sesamin biosynthesis (Kim et al., 2009).

Building on cellular results, *Forsythia* plants stably transformed with sesame CYP81Q1 accumulated sesamin and intermediate piperitol in leaves, though sesamin content was lower than native sesame seeds, indicating further metabolic engineering necessity for yield increases (Koyama et al., 2022a). In a separate study using sesame as the host system, CRISPR/Cas9-mediated downstream enzyme *CYP92B14* knockout in hairy roots reduced sesamin conversion to sesamolin, resulting in substantial sesamin accumulation, demonstrating that blocking endogenous conversion pathways greatly enhances desired metabolite accumulation within natural hosts (see “Potential for Yield Increase” column in **Table 1**) (You et al., 2022).

## Proposed case study: an integrated “Push-Pull-Release” framework for SDG production in flax

Flax represents an attractive model for illustrating an integrated Push-Pull-Release strategy because it is a major dietary source of SDG, its lignan biosynthetic pathway has been relatively well characterized, and hairy root-based transformation systems provide a useful platform for functional analysis and metabolic engineering of phenylpropanoid-derived metabolites (Hano et al., 2006; Markulin et al., 2019). In this context, a multi-layered engineering strategy may be conceptually designed to combine enhanced precursor supply, redirection of competing metabolic flux, and release of endogenous regulatory constraints within a coordinated framework.

For the “Push” component, overexpression of *LuWRKY36*, a flax WRKY TF associated with activation of lignan biosynthetic responses under *Fusarium oxysporum*-elicited conditions, could be considered as a regulatory entry point for stimulating secoisolariciresinol biosynthesis (Markulin et al., 2019). In parallel, co-expression of pathway enzymes such as PLR and a relevant UDP-glycosyltransferase may further promote conversion toward SDG and its stable glycosylated storage form. Alternatively, heterologous expression of a strong phenylpropanoid activator such as AtMYB85 could be considered to enhance monolignol precursor supply, although such an approach would require careful spatial control to avoid excessive whole-plant metabolic perturbation (Kim et al., 2022; Hano et al., 2006).

For the “Pull” component, multiplex CRISPR/Cas9-mediated attenuation of competing phenylpropanoid branches could be used to redirect metabolic flux toward lignan biosynthesis. In particular, disruption of *CHS*, which diverts *p*-coumaroyl-CoA into the flavonoid pathway, and modulation of *F5H*, which contributes to S-lignin biosynthesis, may increase the relative availability of precursor pools that support coniferyl alcohol-derived lignan formation (Cao et al., 2022; Lai et al., 2025). However, because both flavonoid and lignin branches contribute to stress adaptation and structural integrity, such interventions should be regarded as conditional strategies requiring careful phenotypic evaluation rather than universally beneficial modifications (Xie et al., 2018; Ménard et al., 2022).

For the “Release” component, de-repression of phenylpropanoid flux could be pursued by targeting conserved negative regulators of the pathway. For example, suppression of flax orthologs of MYB4-like repressors may alleviate transcriptional constraints on genes such as *PAL*, *4CL*, *CCR*, and *CAD*, whereas disruption of KFB proteins may reduce PAL turnover and thereby increase carbon entry into phenylpropanoid metabolism (Jin et al., 2000; Zhang et al., 2013, 2015). Because the functions of these regulatory modules have been most clearly established in *Arabidopsis* and other model systems, their application in flax should be considered an orthology-based engineering hypothesis requiring direct validation.

From a practical perspective, these “Push”, “Pull”, and “Release” modules could be assembled using modular cloning systems such as Golden Gate or Gibson assembly, either within a stacked construct architecture or through combinatorial transformation designs, depending on cargo size and regulatory complexity (Bird et al., 2022; Gibson et al., 2009). To minimize developmental penalties, pathway activation and de-repression steps would preferably be controlled by tissue-specific or inducible promoters, rather than constitutive expression throughout the plant, thereby restricting metabolic intervention to tissues or developmental stages in which SDG accumulation is desired and structural lignin demand is comparatively lower (Xie et al., 2018). Under such a framework, the anticipated outcome would be increased precursor availability, reduced diversion into competing branches, and prolonged upstream pathway activity, together promoting higher SDG accumulation than could be achieved by any single intervention alone. Rigorous validation would nevertheless be essential. Evaluation should include lignan quantification by high-performance liquid chromatography (HPLC) or liquid chromatography-mass spectrometry (LC-MS) across developmental stages, flavonoid profiling to assess possible trade-offs in stress-protective metabolism, and anatomical or biomechanical analyses to detect unintended effects on lignified tissues arising from perturbation of lignin-related branches (Ménard et al., 2022; Xie et al., 2018). While initial validation can be efficiently conducted in hairy root platforms, these structural characterizations must ultimately be extended to whole-plant regenerants to fully evaluate fitness penalties. Accordingly, this proposed flax case study should be regarded as a rational and testable framework rather than a fully validated engineering blueprint for integrating “Push-Pull-Release” principles in a commercially and nutritionally relevant lignan-producing crop.

## Physiological trade-offs of lignan metabolic engineering in plant fitness

Although the strategies described above can substantially enhance lignan production, their application to the phenylpropanoid network may also affect interconnected pathways involved in structural integrity and disease resistance, underscoring the importance of evaluating potential physiological consequences alongside yield improvements.

### Trade-offs between lignan production and plant structural integrity

A major challenge in lignan metabolic engineering is the competition between lignan biosynthesis and lignin formation for the shared monolignol precursor, CA. Because lignin provides both structural support and hydraulic functionality, diverting flux away from lignin can increase lignan availability but may also weaken xylem biomechanics and water transport. For instance, specific lignin compositions in vascular cells enable tracheary elements to withstand negative pressures, and reduced lignin compromises vascular mechanics and can lead to collapse and impaired stem biomechanics (Ménard et al., 2022). “Pull” strategies that suppress competing lignin branches—such as *F5H* or *4CL* perturbation—can redirect flux within the phenylpropanoid network, but they may also alter cell wall composition and mechanical properties. In poplar, *4CL1* knockout changed lignin composition and triggered compensatory G-lignin biosynthesis at the expense of S-lignin, highlighting the physiological consequences of flux redirection at branch points (Tsai et al., 2020). Flavonoids and lignin share precursor flux within the phenylpropanoid pathway. Suppression of *CHS* to reduce flavonoid production can disturb pathway balance and may affect UV protection and other stress-response traits (Yang et al., 2025). These trade-offs underscore the need for tissue-specific or inducible promoters to enable spatiotemporal control rather than constitutive whole-plant changes (Xie et al., 2018). Future frameworks should integrate cell wall profiling and biomechanical phenotyping with yield assessments to ensure productivity gains do not undermine plant fitness.

### Consequences of lignan metabolic engineering for disease resistance

In addition to structural integrity, lignans and phenylpropanoid intermediates contribute to plant innate immunity and defense signaling, adding complexity to metabolic engineering. Lignans such as pinoresinol and yatein can act directly as antimicrobial compounds. For example, in soybean, the dirigent protein GmDIR22 promotes CA coupling to (+)-pinoresinol, limiting *Phytophthora sojae* hyphal growth, while elevated yatein levels in mycorrhizal plants have been associated with improved resistance to *Botrytis cinerea* (Li et al., 2017; Sanmartín et al., 2020). Similarly, *LTF1* overexpression in *I. indigotica* increased lignan levels and upregulated defense genes alongside biosynthetic genes (Chen et al., 2024). These findings suggest that ERF-type “Push” strategies can strengthen pathogen resistance while maintaining valuable lignan production, although broader trade-offs still need evaluation. However, suppressing competing pathways poses risks because CHS inhibition, a key step for pathogen- and UV-induced flavonoid and isoflavonoid phytoalexin biosynthesis, can reduce antimicrobial flavonoid pools and may compromise UV protection and defense responses (Dao et al., 2011). As a specific instance, *chalcone reductase* (*CHR*) RNAi in soybean decreased daidzein and glyceollin by approximately 90%, severely weakening *P. sojae* resistance (Graham et al., 2007).

“Release” strategies also need to be carefully examined. AtMYB4 represses phenylpropanoid metabolism in part by targeting C4H and modulating sinapate ester production under UV stress; thus, releasing this repression may alter the balance of defense-related phenylpropanoids in ways not yet fully characterized (Jin et al., 2000). *KFB* knockout, which stabilizes PAL and broadly enhances phenylpropanoid biosynthesis, could in principle benefit defense-related metabolite accumulation, but the broader defense and stress-response consequences remain to be systematically evaluated (Zhang et al., 2015). Nevertheless, lignin pathway changes are not always negative. *Caffeoyl-CoA O-methyltransferase* (*CCoA-OMT*) downregulation in alfalfa (*Medicago sativa*) redirected flux toward the isoflavonoid pathway, thereby improving resistance to *F. oxysporum* (Gill et al., 2018). Thus, ERF- and MYB-based “Push” strategies that maintain defense-network integrity are lower-risk than broad knockouts, whereas “Release” interventions require careful characterization of their downstream defense and stress-response effects. Consequently, testing pathogen susceptibility in engineered plants, especially under field-like conditions, should be incorporated into standard practice.

## Conclusion and future perspectives

Plant lignan metabolic engineering has witnessed remarkable progress over recent decades. Early single-enzyme overexpression attempts have advanced to orchestrating entire biosynthetic pathways through transcriptional regulators, culminating in system-level metabolic network redesign using precise CRISPR genome editing technologies. Enhancement strategies evolved beyond simple “pushing” metabolic flux, incorporating “pulling” by blocking competing pathways and “releasing” intrinsic repression mechanisms. Subcellular organelle engineering for spatiotemporal metabolic compartmentalization, combined with synthetic biology modularity principles, opened new avenues for maximizing metabolic efficiency while minimizing host metabolic burden.

Individually, each strategy yields significant gains; future lignan production technology lies in their integrated application. Optimized production systems may integrate, in a combinatorial or stepwise manner: (1) overexpress potent activators like AtMYB85 to globally activate pathways, (2) knock out or attenuate repressors such as MYB4 while also targeting post-translational negative regulators such as KFB proteins to stabilize precursor-supplying enzymes, (3) eliminate competing route enzymes such as CHS and F5H to concentrate precursors toward target pathways, and (4) co-express transporters to rapidly sequester final products into vacuoles. Such multifaceted genetic modifications must be precisely tailored to specific plant species, growth conditions, and target compound properties, necessitating deep species-specific metabolic network and regulatory architecture understanding.

Critically, the analyses of structural integrity and disease resistance trade-offs presented above highlight that maximizing lignan yield cannot be pursued in isolation from plant fitness. Broad suppression of lignin branches risks mechanical and vascular penalties; flavonoid pathway interference undermines innate immunity. Equally, “Release” interventions such as *MYB4* or *KFB* knockout carry their own physiological risks, as broadly de-repressing the phenylpropanoid route can have cascading effects on both cell wall integrity and defense metabolism that remain incompletely characterized.

Future research priorities should therefore focus on three concrete directions. First, spatiotemporally restricted engineering using tissue-specific or stress-inducible promoters must be advanced beyond proof-of-concept toward crop-relevant species, enabling on-demand lignan accumulation without whole-plant metabolic reprogramming. Second, multiplex CRISPR systems capable of simultaneously fine-tuning lignan pathway activators, competing branch enzymes, and structural lignin compensation genes—within a single transformation event—are needed to translate the precision demonstrated in model systems to commercially viable platforms. Third, systematic phenotypic evaluation frameworks encompassing cell wall compositional analysis, biomechanical testing, and standardized pathogen challenge assays should be established as obligatory components of lignan engineering studies, enabling objective assessment of the productivity–fitness balance.

Plant lignan metabolic engineering has achieved striking successes integrating molecular biology, systems biology, and synthetic biology. Future advances in genome-scale metabolic flux modeling and machine learning-guided regulatory circuit design promise establishing powerful, sustainable bio-based platforms for producing high-value lignans that substantially contribute to human health and welfare. These efforts represent a paradigm shift from extractive agriculture toward renewable, engineered production systems for essential pharmaceutical and functional compounds.
